# Trends and inequalities in maternal and child health in a Brazilian city: methodology and sociodemographic description of four population-based birth cohort studies, 1982–2015

**DOI:** 10.1093/ije/dyy170

**Published:** 2019-03-18

**Authors:** Andréa Dâmaso Bertoldi, Fernando C Barros, Pedro R C Hallal, Gregore I Mielke, Paula D Oliveira, Maria Fatima S Maia, Bernardo L Horta, Helen Gonçalves, Aluísio J D Barros, Luciana Tovo-Rodrigues, Joseph Murray, Cesar G Victora, Pelotas Cohorts Study Group, Ana M B Menezes, Alicia Matijasevich, Diego Bassani, Fernando C Wehrmeister, Iná S Santos, Maria Cecilia F Assunção, Mariangela F Silveira, Marlos Rodrigues Domingues

**Affiliations:** 1 Federal University of Pelotas, Brazil; 2 University of São Paulo, Brazil; 3 University of Toronto, Canada; 1 Postgraduate Program in Epidemiology, Federal University of Pelotas, Pelotas, Brazil; 2 Postgraduate Program in Health and Behavior, Catholic University of Pelotas, Pelotas, Brazil; 3 Institute of Human Sciences and Information, Federal University of Rio Grande, Rio Grande, Brazil

**Keywords:** Maternal health, child health, socioeconomic factors, cohort studies, health surveys

## Abstract

**Background:**

Few low-middle-income countries have data from comparable birth cohort studies spanning over time. We report on the methods used by the Pelotas cohorts (1982, 1993, 2004 and 2015) and describe time trends in sociodemographic characteristics of the participant families.

**Methods:**

During the four study years, all maternity hospitals in the city were visited daily, and all urban women giving birth were enrolled. Data on socioeconomic and demographic characteristics were collected using standardized questionnaires, including data on maternal and paternal skin colour, age and schooling, maternal marital status, family income and household characteristics. The analyses included comparisons of time trends and of socioeconomic and ethnic group inequalities.

**Results:**

Despite a near 50% increase in the city’s population between 1982 and 2015, the total number of births declined from 6011 to 4387. The proportion of mothers aged ≥35 years increased from 9.9% to 14.8%, and average maternal schooling from 6.5 [standard deviation (SD) 4.2] to 10.1 (SD 4.0) years. Treated water was available in 95.3% of households in 1982 and 99.3% in 2015. Three-quarters of the families had a refrigerator in 1982, compared with 98.3% in 2015. Absolute income-related inequalities in maternal schooling, household crowding, household appliances and access to treated water were markedly reduced between 1982 and 2015. Maternal skin colour was associated with inequalities in age at childbearing and schooling, as well as with household characteristics.

**Conclusions:**

During the 33-year period, there were positive changes in social and environmental determinants of health, including income, education, fertility and characteristics of the home environment. Socioeconomic inequality was also reduced.

**Key Messages**

- From 1982 to 2015, fertility in Pelotas declined by 44% and, despite a near 50% increase in the city’s population, the number of births declined from 6011 to 4387.
- The proportion of adolescent mothers remained stable at around 15-19%, but the proportion of mothers aged ≥35 years increased from 10 % to 15%.
- There were important improvements in social and environmental determinants of health including family income, parental education and access to safe water.
- Absolute income-related inequalities in most social and environmental conditions improved markedly.
- Black or brown maternal skin colour was associated with inequalities in age at childbearing and schooling, as well as with household characteristics, compared with white women.

## Introduction

Cohort studies have been a vital tool in the development of epidemiology and identification of determinants of health and illness, having served as the basis for many advances in population health by strengthening the evidence base for public health decisions. In particular, there is growing interest in the understanding of how exposures in early life may have long-term consequences for health throughout the life-course,^1^^,^^2^ and birth cohort studies are particularly well suited to test a large number of health hypotheses, especially those involving long latency periods.

There is considerable interest among governments, universities and funding organizations in prospective cohort studies, most of which have taken place in high-income countries (HICs).^3^ Cohort studies in low- and middle-income countries (LMICs) are less common but can make important contributions to this growing literature in several ways. First, LMIC cohorts can investigate risk factors (e.g. intrauterine growth restriction) or outcomes (e.g. infectious diseases) that are rare in HICs. Second, some health exposures differ between settings; for example, physical activity in LMICs is largely related to manual labour and commuting to work, whereas in HICs it is due to leisure-time exercise. Third, remarkably wide socioeconomic differentials are often observed in LMIC cohorts, compared with narrower social gaps in HICs, and this allows a deeper understanding of social determinants of health. And last, confounding factors that are observed in HICs—for example, longer duration of breastfeeding among rich and educated women—may show opposite patterns in LMICs, where breastfeeding is often more prevalent among the poor. Causal inference may be strengthened if results from both types of settings are consistent.^4^ For example, a comparison of the Pelotas 1993 cohort with the British Avon Longitudinal Study of Parents and Children (ALSPAC) showed that associations between breastfeeding and intelligence quotient were similar in spite of different confounding patterns,^5^ indicating a potential cause-effect relationship.

Brazil is the fifth most populous country in the world, with nearly 210 million inhabitants. The country has experienced rapid demographic, economic, nutritional and epidemiological transitions in the recent past, with a huge impact on maternal and child health,^6^ In spite of progress, social inequalities in health have persisted, which is not unexpected given the strong concentration of wealth in a small segment of the Brazilian population,^7^ Recent instability in the economic and political arenas may revert improvements in health that were due to a major reduction in poverty that have occurred since the 1990s.^8^ In addition, the current crisis has had a marked negative impact on health spending as well as on scientific and technological development.^9^

Few if any LMICs have data from comparable, population-based birth cohort studies spanning back over three decades. We report on the four studies carried out in the city of Pelotas in Southern Brazil in 1982, 1993, 2004 and 2015, in which about 20 000 children, adolescents and adults are being followed up since they were born. Although the early Pelotas cohorts focused mostly on child mortality and the consequences of early under-nutrition (in both the short and long term), our interest evolved over time to studying psychomotor development, overweight/obesity and body composition, physical activity, mental health and violence. The evolution of the topics under study followed the health and nutrition transition observed in Brazilian society.^6^ Technical advances also led us to collect information on biomarkers and to study the genomes of cohort members, which was not possible when the first two cohorts were launched.^10^^,^^11^

The four birth cohorts allow comparisons of health, nutrition and human capital indicators over three decades as well as the study of trajectories over time within each cohort. In this article, the first of a supplement, we report the methods used in the four cohorts for documenting time trends in maternal, newborn and infant health over 33 years, as well as for studying how socioeconomic, ethnic and sex inequalities evolved through time. We also describe the socioeconomic and environmental characteristics of the families included in the four cohorts. The remaining articles in the supplement describe characteristics of the mothers (reproductive history, nutritional status, health care during pregnancy and delivery) and of their infants (birthweight and gestational age, mortality, hospital morbidity, infant feeding and nutritional status).

## Methods

Pelotas is a medium sized city in the state of Rio Grande do Sul, located in the south of Brazil (latitude: 31°46’19”, longitude: 2°20’19”), near the Uruguayan border, occupying an area of 1610 km^2^. According to the Brazilian Institute of Geography and Statistics, the municipal population reached 342 873 inhabitants in 2015, of whom 93.3% were urban residents. The main economic activities in Pelotas are agriculture (especially rice production), commerce and education.

From 1 January to 31 December in 1982, 1993, 2004 and 2015, all maternity hospitals in the city were visited daily, and all urban women giving birth were invited to join the study. The city boundaries have changed through time, and an area that was included in the urban perimeter in 1982 was later emancipated as a new municipality (Jardim America). To ensure comparability with the 1982 cohort, women from this neighbourhood were retained in all cohorts.

Primary data collection was conducted using standardized questionnaires to obtain information on socioeconomic, environmental, demographic, nutritional, behavioural and other health-related characteristics. In the 2015 study, we attempted to recruit mothers during antenatal care, to collect prospective information on pregnancy-related variables; 73.8% of the mothers who subsequently delivered children included in the cohort were recruited during antenatal care. All questionnaires are available at [http://www.epidemio-ufpel.org.br/site/content/studies/]. Detailed methodological information on each cohort is available elsewhere.^12–15^

After the perinatal study in hospitals, children were visited at home at different ages. In the 1982 cohort, due to limited funding, the first follow-up visit was carried out in early 1982 and was aimed at the 1916 children born from January to April 1982. Using information on home addresses collected in the perinatal interview, it was possible to locate four-fifths of the intended sample at the mean age of 11.3 months. In face of the low follow-up rate, a new strategy was used in the 2-year follow-up visit during 1984, when a census of all households in the city was carried out and resulted in locating seven out of every eight children born in 1982. For children who had not been traced in 1983, information on the first year of life was obtained retrospectively in 1984; for consistency with the other cohorts, this will be referred to as the 12-month visit. In the 1993 study a subsample of 1460 children, including all low-birthweight children (less than 2500 g) plus a random 20% of the remaining children, were visited at home when infants were aged 1, 3, 6 and 12 months. All children from the 2004 and 2015 were visited at home at the ages of 3 and 12 months. The cohorts continue to be followed up regularly up to the present time, but the analyses in this supplement refer to the first year of life.

Data collection was carried out by trained research team members. Participants were interviewed using standardized, pre-coded questionnaires. The size and complexity of the questionnaires increased substantially between cohorts. In the 1982 perinatal study, 80 questions were printed on the two sides of an A4 sheet. In 1993, the questionnaire comprised 16 pages and 212 questions; and in 2004, the number of pages increased to 25 with 273 questions (some of which were formatted as boxes that included large amounts of information). In 2015, tue questionnaire had 34 pages including 326 questions, and mobile tablets were used instead of paper. In addition to the questionnaire, all babies were weighed and measured. Further details regarding anthropometry and gestational age ascertainment are provided in other articles in this supplement.^16^^,^^17^

To standardize data collection, all team members were trained before each round of fieldwork. The training included general orientations on each question and pre-coded options, and instructions on how to approach the mother and the family in a polite manner. During the follow-up visits, quality control measures included regular calibration of scales and repetition by a supervisor of a subset of interview questions for 5% of the whole sample, including key variables such as age, smoking, education etc. During the fieldwork, interviews were also daily supervised by PhD students.

The analyses reported in the present supplement refer to pregnancies, deliveries and health in the first year of life. Socioeconomic and demographic characteristics were collected during the perinatal interview for the four cohorts: child sex (male, female), maternal and paternal skin colour (white, brown, black), maternal and paternal age in full years, marital status (single or living with partner), maternal and paternal schooling in completed years, family income (expressed in local currency and converted into minimum wages at the time of the perinatal interview), family income in quintiles (with Q1 being the poorest and Q5 the richest) and number of household members. A ‘minimum wage’ is a measure of the legal minimum monthly salary for formal employees in the state.

All analyses relied on data collected from birth to age 12 months, with some exceptions. Treated water supply at home (no, yes) was collected at the 24-month visit in the 1982 cohort, the 6-month follow-up of the 1993 study and during the 3-month follow-up of the 2004 cohort. Presence of a television and refrigerator at home (no, yes) was collected at 24 months for the 1982 cohort and at 6 months for the 1993 study. Paternal schooling was collected at the 12-month visit of the 1982 cohort. The number of household members and of bedrooms were collected when children were 3 months old in the 2004 and 2015 cohorts, but comparable information was not available for the earlier cohorts.

The official Brazilian classification of ethnicity is based on skin colour and includes three frequent categories (white, black or brown) in addition to the less frequent indigenous and yellow (Asian origin) groups. Due to very small number of observations in the Pelotas population, the last two categories were excluded from our analyses of skin colour. In 1982, the interviewer observed the colour of the mother and classified it as white, black or other (either indigenous or yellow); women with brown skin were classified as black. Observation was also used in 1993, but the questionnaire included an additional option for brown skin colour. In 2004 and 2015, the mother self-reported her skin colour according to the five categories, which is currently the standard, widely accepted approach for assessing ethnicity in Brazilian society.^18^

Chi-square statistics were used to test for linear trends over the study period. Inequalities in maternal and household characteristics within each cohort were also analysed using chi-square tests, including tests for trend when appropriate. To evaluate the magnitude of inequalities in maternal and household characteristics, the slope index of inequality and the concentration index were calculated.^19^ The slope index is a measure of absolute inequality, being derived through a logistic regression model. It corresponds to the difference in percentage points between the fitted values of the health indicator for the top and the bottom of the wealth distribution. The concentration index reflects relative inequality and is based on a concept similar to the Gini index for income concentration. It expresses how far from total equality a given distribution is.^20^ Both indices are expressed on a scale from -100 to +100, with zero representing equal distribution of the attribute across the wealth scale. All analyses were conducted with Stata software version 15.0.^21^

Ethical approval for studies was not required in Brazil until 1996. In 1982 and 1993, verbal consent was obtained from the mothers before the interview. The 2004 and 2015 studies were approved by the Ethics Committee of the Federal University of Pelotas, and written consent was obtained from the mothers. Further details on the methods of each cohort are available in previous publications.^10^^,^^11^^,^^13–15^^,^^22–28^

## Results

**Figure 1:**
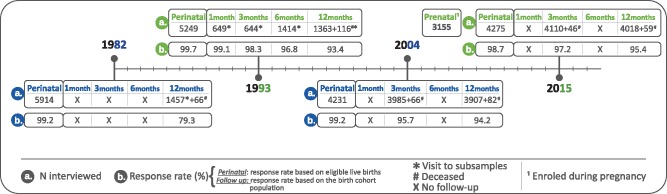
Numbers of live births and of children followed up during the first year of life in the Pelotas birth cohorts, showing response rates.

The total numbers of births in 1982, 1993, 2004 and 2015 were 6011, 5304, 4287 and 4329, respectively. The corresponding numbers of live births were 5914, 5249, 4231 and 4275 (Figure 1), and those of singleton live births 5816, 5168, 4147, 4164, respectively. Refusal rates at the perinatal interview, when all urban women giving birth were invited, were 1.3% or less. Response rates at the 12-month interview were 79% in 1982 and above 93% for the 1993, 2004 and 2015 cohorts. In the 1982 cohort, a census of all households in the city increased the follow-up rate to 87.2% at the age of 2 years, when retrospective information on the first year of life was obtained. Figure 1 describes the timelines of the first year of each birth cohort, with response rates.

Table 1 shows characteristics of Pelotas over the time span of the cohort studies. The city population increased from nearly 250 000 to around 340 000 between 1982 and 2015. In 1982 almost a fifth of population was rural; this proportion decreased to 6.7% in 2015. The ratio between the gross domestic product (GDP) per capita in Pelotas and Brazil is also shown in Table 1. In 1982 Pelotas had a slight higher GDP per capita than Brazil, with a ratio of 1.09, but this ratio decreased to 0.74 by 2015, showing a relative impoverishment of the city. The number of hospitals, and in particularly of hospital beds, was substantially reduced, particularly due to the shift in psychiatric care from inpatient to outpatient care. The number of public sector primary care units increased from 37 to 54. Of particular interest for the cohorts is the fact that whereas in 1982 there were no neonatal or paediatric intensive care units, 16 beds were available in 2015, 19 in 2004 and 29 in 2015.

**Table 1.** Characteristics of Pelotas-RS, Brazil, over the cohort studies (1982–2015)

Characteristics	1982	**1993**^l^	2004	2015
Total population (thousands)^a^	250	300	335	343
% living in the urban area^a^	81.0	91.6	93.2	93.3
Municipal Human Development Index^b^	–	0.558^c^	0.660^d^	0.739^e^
Crude birth rate (births/1000 population)^f^	23.1^g^	19.3^h^	13.3	12.9
Gross Domestic Product per capita (US$)^i^	3989	–	2511	5953
Ratio Pelotas/Brazil: Gross Domestic Product^i^	1.09	–	0.61	0.74^j^
Gini index for income distribution^b^	–	0.59^a^	0.60^b^	0.56^c^
Brazilian monthly minimum wage (US$)^i^	77.4	31.4	88.9	236.9
Number of maternity hospitals	3	5	5	5
Number of hospital beds	1577	1431	1321	1169
Beds in paediatric intensive care units^k^	0	16	19	29
Primary health care units	37	59	59	54

a Estimates were derived through interpolation of the results of the 1980, 1991, 2000 and 2010 national censuses, and projections for 2015. Source: Brazilian Institute of Geography and Statistics (IBGE) [https://ww2.ibge.gov.br/home/estatistica/populacao/estimativa2015/estimativa_dou.shtm].

b Atlas of Human Development in Brazil [http://www.atlasbrasil.org.br/2013/ranking].

c Official data available for 1991, Atlas of Human Development in Brazil [http://www.atlasbrasil.org.br/2013/ranking].

d Official data available for 2000—Atlas of Human Development in Brazil—http://www.atlasbrasil.org.br/2013/ranking.

e Official data available for 2010, Atlas of Human Development in Brazil [http://www.atlasbrasil.org.br/2013/ranking].

f Estimate based on Brazilian Institute of Geography and Statistics and Live Birth Information System (SINASC) [http://tabnet.datasus.gov.br/cgi/tabcgi.exe? ibge/cnv/pibmunbrs.def].

g Non-official data. Estimate based on number of live births from 1982 Cohort.

h Official data available for 1994. Estimate based on SINASC.

i Fundação de Economia e Estatística [http://www.fee.rs.gov.br].

j Official data available for 2014. Source: Fundação de Economia e Estatística [http://www.fee.rs.gov.br].

k Paediatric and neonatal intensive care beds.

l Economic data for 1993 should be interpreted with caution due as hyperinflation was taking place during this year (annual inflation ratio of 2477.2%).

There were major changes in the organization of health services in Brazil and in Pelotas throughout the study period. In 1982, services presented the tradition three-tier system that was common in Latin America: private care, the National Social Security Institution for regularly employed citizens and their families, and the indigent category. The latter included the poorest strata of the population who could not afford private care and were not regularly employed; it included 5.6% of all women in the 1982 cohort. In 1988, the National Health System was created, a tax-based universal system that covers the whole population. Yet substantial proportions of families, although entitled to the National Health System, prefer to take out private insurance where care is perceived as being better, and access to consultations and examinations is faster. In 2015, 45% of the women studied belonged to the private insurance system; it should be noted that many of these private plans do not cover for hospital admissions, so that 68% of all women who gave birth were covered by the National Health Service. The Supplementary Table, available as Supplementary data at *IJE* online shows the proportions of births in the four cohorts according to hospital and type of payment for delivery.

Table 2 presents trends in child, maternal, paternal and household characteristics from 1982 to 2015. Except for 1993, more boys than girls were born. There was a constant decline in the proportion of white mothers, from 82.1% in 1982 to 71.9% in 2015. A similar decline was observed for paternal skin colour for the three cohorts when this information was collected. The proportions of single mothers increased from 8.2% to 14.2%, respectively.

**Table 2.** Sample characteristics according to birth cohort

Variables	Cohort perinatal sample, *n* (%)				
	1982	1993	2004	2015	*P*
Sex					0.948
Males	3037 (51.4)	2603 (49.6)	2196 (51.9)	2164 (50.6)	
Females	2876 (48.6)	2645 (50.4)	2035 (48.1)	2111 (49.4)	
Maternal skin colour					<0.001
White	4851 (82.1)	4058 (77.3)	3090 (73.0)	3071 (71.9)	
Brown	–	234 (4.5)	295 (7.0)	561 (13.1)	
Black	1060 (17.9)^c^	955 (18.2)	846 (20.0)	639 (15.0)	
Paternal skin colour					<0.001
White	–	4064 (77.9)	2709 (66.3)	2983 (71.4)	
Brown	–	256 (4.9)	793 (19.4)	541 (13.0)	
Black	–	899 (17.2)	583 (14.3)	653 (15.6)	
Marital status					<0.001
Single mother	485 (8.2)	649 (12.4)	695 (16.4)	607 (14.2)	
With partner	5424 (91.8)	4600 (87.6)	3536 (83.6)	3667 (85.8)	
Maternal age (years)					<0.001
12–19	912 (15.4)	915 (17.4)	800 (18.9)	622 (14.6)	
20–24	1843 (31.2)	1447 (27.6)	1149 (27.2)	1011 (23.6)	
25–29	1599 (27.0)	1353 (25.8)	959 (22.7)	1006 (23.5)	
30–34	973 (16.5)	956 (18.2)	758 (17.9)	1003 (23.5)	
≥35	586 (9.9)	577 (11.0)	563 (13.3)	632 (14.8)	
Paternal age (years)					<0.001
13–19	–	302 (5.8)	308 (7.4)	256 (6.1)	
20–24	–	1194 (23.1)	970 (23.4)	843 (20.1)	
25-29	–	1340 (25.9)	994 (24.0)	901 (21.5)	
30-39	–	1815 (35.1)	1366 (33.0)	1703 (40.5)	
≥40	–	517 (10.0)	503 (12.2)	498 (11.9)	
Maternal schooling (years)					<0.001
<4	1282 (21.7)	832 (15.9)	348 (8.3)	173 (4.1)	
4-8	3132 (53.0)	3060 (58.4)	2038 (48.7)	1313 (30.7)	
9-11	654 (11.1)	923 (17.6)	1382 (33.0)	1458 (34.1)	
≥12	839 (14.2)	427 (8.2)	420 (10.0)	1330 (31.1)	
Paternal schooling (years)					<0.001
<4	266 (19.5)	732 (15.1)	318 (9.7)	213 (5.3)	
4–8	727 (53.4)	2863 (59.0)	1423 (43.3)	1469 (36.9)	
9-11	174 (12.8)	908 (18.7)	1181 (35.9)	1258 (31.6)	
≥12	194 (14.3)	352 (7.3)	364 (11.1)	1043 (26.2)	
Family income (minimum wages)					<0.001
≤1	1288 (21.9)	967 (18.8)	897 (21.2)	538 (12.6)	
>1–3	2789 (47.4)	2148 (41.8)	1939 (45.8)	2014 (47.1)	
>3–6	1091 (18.5)	1204 (23.4)	945 (22.3)	1127 (26.4)	
>6-10	382 (6.5)	433 (8.4)	243 (5.7)	324 (7.6)	
>10	335 (5.7)	385 (7.5)	207 (4.9)	270 (6.3)	
Family income (quintiles)					0.867
Q1 (poorest)	1183 (20.0)	1031 (20.1)	872 (20.6)	846 (19.8)	
Q2	1178 (19.9)	1195 (23.3)	855 (20.2)	859 (20.1)	
Q3	1180 (20.0)	889 (17.3)	816 (19.3)	853 (20.0)	
Q4	1185 (20.0)	1001 (19.5)	858 (20.3)	856 (20.0)	
Q5 (richest)	1188 (20.1)	1021 (19.9)	830 (19.6)	859 (20.1)	
Household members					<0.001
≤3	–	–	1197 (30.0)	1545 (37.7)	
4–5	–	–	1943 (48.8)	1990 (48.5)	
≥6	–	–	845 (21.2)	569 (13.9)	
Household crowding (persons/bedroom^a^)					<0.001
≤2	–	–	1250 (31.4)	1678 (40.9)	
>2	–	–	2735 (68.6)	2425 (59.1)	
Treated water					<0.001
No	234 (4.7)	49 (3.5)	39 (1.0)	33 (0.8)	
Yes^b^	4757 (95.3)	1365 (96.5)	3945 (99.0)	4240 (99.2)	
Television					<0.001
No	668 (13.3)	183 (12.8)	163 (3.9)	34 (0.8)	
Yes	4338 (86.7)	1231 (87.2)	4066 (96.2)	4236 (99.2)	
Refrigerator					<0.001
No	1235 (24.7)	375 (25.7)	458 (10.8)	72 (1.7)	
Yes	3771 (75.3)	1039 (74.3)	3771 (89.2)	4198 (98.3)	
Total	5914	5249	4231	4275	

a Number of bedrooms = bedrooms used for sleeping.

b Treated water = source of piped water indoors or on the ground.

c Brown and black combined in 1982.

*P*-value: χ² test for trend.

Overall, during the 33-year period, mothers became older, although the proportion of adolescent mothers remained stable at around 15%. The percentage of mothers aged ≥30 years increased from about 25% in 1982 to almost 40% in 2015. Paternal age also increased from 1993 to 2015. Maternal schooling increased considerably; mean values were 6.5 (SD 4.2) years in 1982, 6.7 (SD 3.6) in 1993, 8.1 (SD 3.5) in 2004 and 10.1 (SD 4.0) in 2015. The percentage of mothers with <4 years of schooling declined from 21.7% in 1982 to 4.1% in 2015. Conversely, the proportion with ≥12 years of schooling increased from 14.2% in 1982 to 31.1% in 2015. Similar patterns were documented for paternal schooling, with the proportion with <4 years falling from 19.5% to 5.3%, respectively. The proportion of families earning one or less minimum wage per month declined from 21.9% in 1982 to 12.6% in 2015. The division of the sample into quintiles naturally resulted in roughly equal groups in all cohorts, each with about 20% of the sample (these numbers are presented because they provide the denominators for several analyses in other articles in this supplement). Household characteristics also changed considerably over time. Information on crowding was available for 2004 and 2015, showing a reduction from 21.2% to 13.9% in families with six or more members, and a corresponding reduction in the proportion of homes with more than two persons per bedroom. Treated water was available in 95.3% of households in 1982 and in 99.28% in 2015. Three-quarters of the families had a refrigerator at home in 1982, compared with 98.3% in 2015, and television ownership became practically universal.

Involvement of the women in the labour market became more common with time. In the 1982 cohort, 26.2% of the women worked in the formal or informal sector during the child’s first year of life. This proportion increased 37.1% in 1993, 39.4% in 2004 and 48.3% in 2015.

Income-related inequalities in maternal age, schooling, household size, appliances and access to treated water are presented in Table 3, which also shows the summary indices for absolute (slope index) and relative (concentration index) inequalities. Both indices range from -100 to +100, with zero representing complete equality. Negative values indicate that the outcome is more frequent among the poor, whereas positive indices indicate higher frequency among the rich. Teenage mothers were proportionately about five times more common in the poorest than in the richest quintile in 1982, and six times more common in 2015, so that inequalities increased slightly over time. In contrast, the proportion of mothers aged ≥35 years remained stable in the poorest quintile but more than doubled in the richest, leading to sharp increases in inequality. Income-related gaps in low maternal schooling (<4 years) were markedly reduced in absolute, but not in relative terms. Absolute inequalities in television and refrigerator ownership, and in having untreated water, also fell markedly.

**Table 3.** Maternal and household characteristics according to quintiles of family income

	Family income (quintiles) % (95% CI)							
	Q1	Q2	Q3	Q4	Q5	*P*-value^a^	Slope index of inequality (95% CI)	Concentration index (95% CI)
Maternal age ≤19 years, *P*	0.330	0.001	0.002	0.176	0.446			
1982	26.1 (23.6−28.6)	15.4 (13.3−17.4)	18.9 (16.7−21.1)	11.1 (9.3−12.9)	5.6 (4.3−7.0)	<0.001	−22.4 (−25.6; −19.3)	−23.7 (−26.9; −20.6)
1993	22.0 (19.5−24.6)	22.6 (20.2−25.0)	15.6 (13.2−18.0)	16.9 (14.6−19.2)	8.0 (6.4−9.7)	<0.001	−15.1 (−18.6; −11.6)	−13.7 (−17.1; −10.4)
2004	28.3 (25.3−31.3)	24.3 (21.4−27.2)	20.0 (17.2−22.7)	13.1 (10.8−15.3)	8.6 (6.6−10.5)	<0.001	−25.0 (−29.0; −21.0)	−21.7 (−25.0; −18.5)
2015	26.4 (23.4−29.3)	20.8 (18.1−23.6)	12.0 (9.8−14.1)	9.6 (7.6−11.6)	4.2 (2.8−5.5)	<0.001	−27.8 (−31.5; −24.0)	−30.3 (−34.0; −26.6)
Maternal age ≥35 years, *P*	0.404	0.599	<0.001	<0.001	<0.001			
1982	8.0 (6.4−9.5)	12.5 (10.6−14.4)	7.2 (5.7−8.7)	9.7 (8.0−11.4)	12.2 (10.3−14.1)	0.036	2.8 (1.4; 5.5)	5.3 (0.8; 9.7)
1993	7.1 (5.5−8.7)	8.5 (6.9−10.0)	13.0 (10.8−15.3)	11.2 (9.2−13.1)	16.3 (14.0−18.5)	<0.001	9.7 (6.7; 12.6)	14.6 (10.3; 18.8)
2004	8.9 (7.0−10.7)	11.7 (9.5−13.9)	13.0 (10.7−15.3)	14.2 (11.9−16.6)	19.0 (16.4−21.7)	<0.001	11.3 (7.7; 14.9)	13.4 (9.0; 17.8)
2015	8.5 (6.6−10.4)	10.8 (8.7−12.9)	12.5 (10.3−14.8)	15.8 (13.3−18.2)	26.2 (23.3−29.2)	<0.001	19.9 (16.1; 23.8)	21.3 (17.2; 25.4)
Maternal schooling <4 years, *P*	<0.001	<0.001	<0.001	<0.001	<0.001			
1982	46.2 (43.3−49.0)	41.6 (38.8−44.4)	10.2 (8.4−11.9)	8.5 (6.9−10.1)	2.1 (1.3−2.9)	<0.001	−59.0 (−62.1; −56.0)	−44.1 (−46.2; −42.1)
1993	31.4 (28.6−34.2)	18.4 (16.2−20.6)	13.8 (11.5−16.0)	9.0 (7.2−10.8)	4.3 (3.0−5.6)	<0.001	−28.6 (−32.3; −25.0)	−29.2 (−32.6; −25.8)
2004	16.1 (13.7−18.6)	11.6 (9.4−13.7)	8.0 (6.1−9.9)	4.3 (3.0−5.7)	1.0 (0.2−1.5)	<0.001	−19.5 (−22.7; −16.4)	−36.7 (−41.3; −32.0)
2015	9.5 (7.5−11.4)	5.8 (4.3−7.4)	3.4 (2.2−4.6)	1.4 (0.6−2.2)	0.2 (0.0−0.6)	<0.001	−12.2 (−14.7; −9.6)	−48.1 (−54.1; −42.0)
Number of household members ≥6, *P*	<0.001	<0.001	<0.001	0.003	<0.001			
1982	–	–	–	–	–		–	–
1993	–	–	–	–	–		–	–
2004	26.5 (23.3−29.5)	24.8 (21.8−27.7)	20.8 (18.0−23.7)	18.8 (16.1−21.4)	15.1 (12.5− 17.6)	<0.001	−14.1 (−18.5; 9.8)	−10.0 (−13.5; −6.6)
2015	17.9 (15.3−20.6)	16.4 (13.9−19.0)	12.8 (10.5−15.0)	13.3 (11.0−15.7)	8.8 (6.9−10.8)	<0.001	−10.3 (−14.0; −6.7)	−12.3 (−16.7; −8.0)
Families with no television, *P*	<0.001	<0.001	<0.001	<0.001	0.009			
1982	33.9 (30.9−37.0)	16.7 (14.4−19.1)	11.2 (9.2−13.1)	5.4 (4.1−6.8)	1.0 (0.4−1.6)	<0.001	−39.0 (−42.5; −35.5)	−44.2 (−47.3; −41.2)
1993	31.2 (25.8−36.6)	15.0 (11.2−18.8)	10.3 (6.5−14.0)	3.8 (1.5−6.2)	1.6 (0.0−3.1)	<0.001	−35.4 (−42.2; −28.7)	−42.3 (−49.3; −36.5)
2004	9.9 (7.9−11.8)	4.8 (3.4−6.2)	2.8 (1.7−4.9)	1.3 (0.5−2.0)	0.2 (0.0−0.5)	<0.001	−12.4 (−15.1; −9.8)	−49.0 (−55.2; −42.8)
2015	1.2 (0.5−1.9)	1.3 (0.5−2.0)	0.6 (0.0−1.1)	0.7 (0.1−1.3)	0.2 (0.0−0.6)	0.010	−1.2 (−2.2; −0.1)	−24.3 (−41.9; −6.7)
Families with no refrigerator, *P*	<0.001	<0.001	<0.001	<0.001	<0.001			
1982	56.3 (53.2−59.5)	35.1 (32.1−38.1)	22.9 (20.3−25.5)	9.2 (7.4−10.9)	2.3 (1.4−3.3)	<0.001	−62.6 (−65.7; −59.6)	−42.3 (−44.4; −40.2)
1993	55.1 (49.3−60.9)	32.0 (27.0−36.9)	22.1 (17.0−27.3)	13.8 (9.6−18.1)	4.8 (2.1−7.4)	<0.001	−55.3 (−61.8; −48.8)	−34.5 (−38.9; −30.1)
2004	27.2 (24.3−30.2)	15.3 (12.9−17.7)	7.5 (5.7−9.3)	2.4 (1.4−3.5)	1.0 (0.3−1.6)	<0.001	−35.4 (−39.1; −31.6)	−48.5 (−52.0; −45.0)
2015	3.8 (2.5−5.1)	2.2 (1.2−3.2)	1.2 (0.4−1.9)	0.8 (0.2−1.4)	0.5 (0.0−0.9)	<0.001	−4.1 (−5.7; −2.5)	−34.9 (−46.4; −23.4)
Untreated water, *P*	<0.001	<0.001	<0.001	<0.001	0.666			
1982	11.1 (9.1−13.1)	6.6 (5.1−8.1)	3.8 (2.6−5.0)	2.0 (1.2−2.9)	0.5 (0.0−0.9)	<0.001	−13.3 (−15.8; −10.8)	−41.5 (−47.1; −36.0)
1993	9.1 (5.8−12.5)	3.2 (1.3−5.1)	2.8 (0.7−4.8)	1.2 (0.0−2.5)	0.4 (0.0−1.2)	<0.001	−9.9 (−14.4; −5.5)	−46.0 (−59.5; −32.5)
2004	2.3 (1.3−3.4)	1.4 (0.6−2.2)	0.9 (0.2−1.6)	0.1 (0.0−0.4)	0.1 (0.0−0.4)	<0.001	−2.9 (−4.2; −1.7)	−48.6 (−61.9; −35.3)
2015	0.7 (0.1−1.3)	1.7 (0.9−2.6)	0.7 (0.1−1.3)	0.2 (0.0−0.6)	0.5 (0.0−0.9)	0.034	−1.0 (−1.9; −0.1)	−22.6 (−40.1; −5.1)

*P*-values for inter-cohorts chi-square test for trend.

a *P*-values for intra-cohort chi-square test for trend.

Table 4 shows trends in maternal and household characteristics according to maternal skin colour. The proportion of adolescent mothers was higher among black or brown mothers in all years except for 2004, but the proportion of mothers aged ≥35 years only increased over time among Whites. Low maternal schooling (<4 years) was reduced in all skin colour groups, but differences were still marked as of 2015. Households with six or more members were twice as common for black or brown compared with for white women in the two latest cohorts, when data were available. Gaps in television and refrigerator ownership and in access to treated water were eliminated as universal coverage was reached.

**Table 4.** Maternal and household characteristics according to skin colour

	Skin colour % (95% CI)			
	Black	Brown	White	*P*-value^a^
Maternal age ≤ 19 years, *P*	0.127*		0.100	
1982	17.4 (15.1-19.6)		15.0 (14.0-16.0)	0.055
1993	20.3 (17.8-22.9)	20.9 (15.7-26.1)	16.6 (15.4-17.7)	0.008
2004	19.3 (16.6-21.9)	18.3 (13.9-22.7)	18.9 (17.5-20.3)	0.931
2015	20.3 (17.2-23.5)	20.9 (17.5-24.2)	12.2 (11.0-13.4)	<0.001
Maternal age ≥35 years, *P*	0.376*		<0.001	
1982	11.1 (9.2-13.0)		9.6 (8.8-10.5)	0.143
1993	10.9 (8.9-12.9)	10.3 (6.4-14.2)	11.1 (10.1-12.0)	0.922
2004	12.2 (10.0-14.4)	15.3 (11.1-19.4)	13.4 (12.2-14.6)	0.376
2015	13.0 (10.4-15.6)	10.2 (7.7-12.7)	16.0 (14.7-17.3)	0.001
Maternal schooling <4 years, *P*	<0.001*		<0.001	
1982	34.1 (31.2-36.9)		19.0 (17.9-20.1)	<0.001
1993	23.2 (20.6-26.0)	24.4 (18.8-29.9)	13.6 (12.6-14.7)	<0.001
2004	12.2 (10.0-14.4)	11.9 (8.2-15.7)	6.9 (6.0-7.8)	<0.001
2015	8.8 (6.6-11.0)	6.2 (4.2- 8.2)	2.7 (2.1-3.2)	<0.001
Number of household members ≥6, *P*	<0.001*		<0.001	
1982	–		–	–
1993	–	–	–	–
2004	34.4 (31.2-37.8)	19.6 (15.0-24.3)	17.8 (16.4-19.2)	p < 0.001
2015	24.2 (20.8-27.6)	18.9 (15.6-22.2)	10.8 (9.7-11.9)	p < 0.001
Families with no television, *P*	<0.001*		<0.001	
1982	23.6 (20.8-26.4)		11.1 (10.1-12.1)	<0.001
1993	20.2 (16.4-28.1)	20.7 (10.3-31.1)	10.3 (8.3-12.2)	<0.001
2004	5.7 (4.1-7.2)	5.8 (3.1-8.4)	3.2 (2.6-3.8)	0.001
2015	0.6 (0.0-1.2)	1.1 (0.2-1.9)	0.7 (0.4- 1.1)	0.656
Families with no refrigerator, *P*	<0.001*		<0.001	
1982	42.2 (38.9-45.4)		20.9 (19.6-22.1)	<0.001
1993	38.9 (32.1-46.7)	40.9 (28.4-53.5)	22.0 (19.3-24.6)	<0.001
2004	18.0 (15.4-20.6)	18.6 (14.2-23.1)	8.1 (7.2-9.1)	<0.001
2015	2.5 (1.3-3.7)	2.0 (0.8-3.1)	1.5 (1.0-1.9)	0.156
Untreated water, *P*	<0.001*		<0.001	
1982	6.8 (5.1-8.4)		4.2 (3.6-4.8)	0.001
1993	2.4 (0.4-4.3)	10.8 (2.7-18.9)	3.2 (2.1-4.3)	0.057
2004	1.4 (0.6-2.2)	2.5 (0.7- 4.3)	0.7 (0.4-1.0)	0.006
2015	0.5 (0.0-1.0)	0.9 (0.1-1.7)	0.8 (0.5-1.1)	0.626

*P*-values for inter-cohorts chi square test for trend.

a *P*-values for intra-cohort chi-square test.

* *P*-values for black or brown combined into a single category in 1993, 2004 and 2015, allowing comparison with 1982.

## Discussion

The present article is the first in a series of 10 publications reporting on time trends and inequalities in indicators related to pregnancy, delivery and the first year of life in four birth cohorts spanning 33 years. Our series is one of the few in world with prospective, population-based data collection using similar methods over such a long period of time. The existence of four cohorts allows longitudinal analyses of developmental origins of health and disease, as well as comparisons of how maternal and child indicators have evolved over time. The present supplement is focused on the second type of analysis, with special attention to wealth-related and ethnic group inequalities, which have been and remain key drivers of health conditions in Brazil, one of the least egalitarian countries in the world.^29^

The cohorts span a period of rapid transformation in Brazilian society, with positive trends consisting of a reduction in poverty and in fertility, massive declines in infectious diseases and in infant mortality, urbanization and the creation of a national health service (the Sistema Único de Saúde or SUS).^7^ Many of these trends are reflected in the sociodemographic characteristics described here. These include important increases in parental education, family income and availability of safe water and household appliances. Reduced fertility led to smaller families and to a marked drop in the city birth rate, from 23 to 13 births per thousand inhabitants over the 33-year period. This was accompanied by an important increase in the percentage of mothers aged ≥35 years, as childbearing was postponed among the rich. Poverty reduction was part of a national trend; the inflation-adjusted value of the minimum wage increased, and at the same time the proportion of families earning less than one minimum wage declined. It should be noted, however, that poverty reduction in Pelotas was not as rapid as for the country as a whole. Whereas in 1982 the city’s gross domestic product was 9% above the national mean, by 2015 it was 26% lower (Table 1). This may explain why local improvements in the health of mothers and children were not, in some instances, as marked as those observed for the rest of Brazil. Such comparisons will be presented in the next articles in this supplement.

Since 1988, the Pelotas cohorts have had a strong focus on health inequalities.^30^ The comparison of the four cohorts shows that absolute disparities associated with family wealth were greatly reduced over time for characteristics such as parental schooling and household conditions. For some indicators where prevalence was close to zero in the richest quintile—such low education, untreated water or lack of television or refrigerator—the declines in absolute inequalities were not always consistent with declines in relative inequalities, as the latter are highly sensitive to low values in the better-off group. Such apparent paradoxical results are not unusual in the literature on time trends in inequalities. This is why it is important to report on both absolute and relative inequalities, and to allow readers to reach their own interpretation.^31^ In contrast to indicators for which inequalities declined, the proportion of teenage mothers according to income groups became slightly less equitable over time with an increase among the poor, whereas the proportion of mothers aged ≥35 years remained stable among the poor but increased sharply among the rich, reflecting delayed childbearing in the latter group, likely associated with educational achievements and career choices.^32^

We also focused on ethnic group inequalities. The main economic activity in Pelotas in the 1800s was the manufacture of sun-dried beef or ‘jerky’. Cattle from the Pampas region were brought to Pelotas where they were slaughtered and their meat was dried, and then shipped to Rio de Janeiro, São Paulo and other populous areas in Brazil. African slaves were brought to Pelotas in large numbers to provide the intense manual labour involved in this industry.^33^

As a consequence, Pelotas is one of the cities in Southern Brazil with the highest proportion of Afro-descendants, who in the 2015 cohort represented almost 30% of all women who gave birth. Because of the marked miscegenation that characterizes our population, the proxy for ethnicity used in national censuses and surveys is self-reported skin colour. This classification is endorsed by the black movement, which advocates for disaggregation of all relevant national statistics in order to raise the visibility of Afro-descendants.^18^ In our cohorts, the proportion of brown or black women giving birth increased from 18% to 28% over time. It is unclear whether this was due to changes in the way this variable was ascertained (according to the interviewer in 1982 and 1993, and through self-report in 2004 and 2015), to differential fertility rates over time, and/or to increased visibility of the black movement leading to greater recognition of African ancestry.

Despite the methodological limitations associated with measurement of ethnicity over the four cohorts, disparities are evident. For example, adolescent childbearing, low maternal schooling and household crowding are more prevalent among Afro-descendants, whereas late childbearing prevails among Whites. These findings justify the need for disaggregating health statistics according to skin colour, and for designing public policies that allow Afro-descendant women and children the same living conditions and access to education and health that is enjoyed by white women and children.

It is important to highlight that the data presented here are not necessarily representative of Brazil as a whole. However, the present findings could have important implications for the country and other middle-income countries facing demographic transition, and where public and private sectors coexist. To assess the external validity of our cohort findings, we have a strong collaboration with other cohorts from low- and middle-income countries (Guatemala, India, the Philippines and South Africa) which has generated tens of publications on the long-term consequences of under-nutrition,^34^ as well as with other Brazilian cohorts.^35^ We have also collaborated with cohorts from high-income countries, including the United Kingdom, The Netherlands and Belarus,^5^^,^^36^ mainly in order to improve causal inference through cross-cohort comparisons, and joined multisite studies on genetic epidemiology.^37–40^

One limitation of this study, which is common in birth cohorts, has been attrition rates in the follow-up visits. However, except for the 12-month visit of the 1982 cohort, in which we could not trace 20.7% of the children, we located at least 90% of all children in all other visits. Losses in 1982 were more frequent among the poorest and the richest strata of the population, as middle-class families were more easily found.^25^ Another limitation inherent in this type of study is that data collection is based primarily on self-reports, mostly from the mother during the life period covered in this article. Given changes in medical practice over time, gestational age was ascertained with different methods in the early and late cohorts, and there were also differences in how weight at the end of the pregnancy was measured; these discrepancies are described in the articles on specific outcomes in this supplement.

Thus, data on morbidity during pregnancy, antenatal care, labour induction, infant morbidity etc. are based on what is stated by the interviewee. The quality of this information is variable and depends on characteristics relating to the interviewee (such as age and schooling), and on the type of information (personal, medical). The possibility of information error affecting the results of specific analyses is discussed in each article. On the other hand, the cohort strategy allows for this information to be collected close to its occurrence, thus minimizing recall bias.

Yet another limitation is that the 1993 cohort study took place during a period of hyperinflation. According to the National Consumer Price Index, whereas annual inflation in 1982 was 104.8%, 7.6% in 2004 and 10.7% in 2015, in 1993 annual it reached 2477.2%, which may introduce noise in the income data collected in that year.^41^ Hyperinflation ended in mid-1994, when a new economic plan was introduced and turned the currency into ‘Real’, which is still in use in the country.^42^

In the present article, we provide background information on the methodology of the four Pelotas cohorts and a general description of sociodemographic and environmental conditions of the families included, with emphasis on social and ethnic inequalities. The information presented here will contribute to the interpretation of time trends and disparities in maternal and child health outcomes, which will be presented in the following eight articles included in the supplement.

## Funding

The four cohorts received funding from the following agencies: Wellcome Trust, International Development Research Center, World Health Organization, Overseas Development Administration of the United Kingdom, European Union, Brazilian National Support Program for Centers of Excellence (PRONEX), Brazilian National Council for Scientific and Tehcnological Development (CNPq), Science and Technology Department (DECIT) of the Brazilian Ministry of Health, Research Support Foundation of the State of Rio Grande do Sul (FAPERGS), Brazilian Pastorate of the Child and Brazilian Association for Collective Health (ABRASCO).

## Supplementary Material

Supplementary Table
