# Supplementary material for: Trends and inequalities in maternal and child health in a Brazilian city: methodology and sociodemographic description of four population-based birth cohort studies, 1982–2015
Source: Int J Epidemiol. 2019 Mar 18;48(Suppl 1):i4–i15. doi: 10.1093/ije/dyy170 (PMC6422064; doi:10.1093/ije/dyy170)
Supplement: Supplementary Table [file dyy170_supplementary_table.pdf]

Proportions of births in the four cohorts according to hospital and type of payment for delivery. Pelotas, 1982-2015.

			Hospital name				
			Santa Casa	São Francisco de Paula	Beneficência	Fundação de Apoio Universitário	Miguel Piltcher
1982 cohort	**Proportion of all births in the city**		**39,1%**	**18,6%**	**42,2%**	**-**	-
	Type of payment for delivery	Social security	71,8%	86,5%	85,2%	-	-
		Private insurance	6,4%	2,3%	5,4%	-	-
		Out of pocket	7,9%	11,2%	9,4%	-	-
		Charity	13,9%	0,0%	0,0%	-	-
1993 cohort	**Proportion of all births in the city**		**35,8%**	**27,4%**	**29,5%**	**4,8%**	**1,7%**
	Type of payment for delivery	Social security	89,0%	90,4%	82,3%	99,2%	2,2%
		Private insurance	6,7%	5,1%	11,3%	0,4%	85,7%
		Out of pocket	4,3%	4,5%	6,3%	0,4%	12,1%
2004 cohort	**Proportion of all births in the city**		**36,4%**	**30,8%**	**5,3%**	**23,1%**	**3,9%**
	Type of payment for delivery	Social security	87,3%	83,6%	0,5%	100,0%	0
		Private insurance	10,0%	12,9%	83,0%	0,0%	65,4%
		Out of pocket	2,7%	3,4%	16,5%	0,0%	34,6%
2015 cohort	**Proportion of all births in the city**		**35,6%**	**35,1%**	**1,3%**	**18,6%**	**9,2%**
	Type of payment for delivery	Social security	79,6%	60,0%	0,0%	100,0%	0,3%
		Private insurance	7,8%	30,7%	78,6%	0,0%	75,5%
		Out of pocket	12,6%	9,3%	21,4%	0,0%	24,2%
